# Integrated analysis of differentially expressed genes and construction of a competing endogenous RNA network in human Huntington neural progenitor cells

**DOI:** 10.1186/s12920-021-00894-2

**Published:** 2021-02-12

**Authors:** Xiaoping Tan, Yang Liu, Taiming Zhang, Shuyan Cong

**Affiliations:** Department of Neurology, Shengjing Hospital of China Medical University, Shenyang, 36 Sanhao Street, Shenyang, 110004 Liaoning People’s Republic of China

**Keywords:** RNA, lncRNA, Huntington disease, ceRNA, Bioinformatics

## Abstract

**Background:**

Huntington's disease (HD) is one of the most common polyglutamine disorders, leading to progressive dyskinesia, cognitive impairment, and neuropsychological problems. Besides the dysregulation of many protein-coding genes in HD, previous studies have revealed a variety of non-coding RNAs that are also dysregulated in HD, including several long non-coding RNAs (lncRNAs). However, an integrated analysis of differentially expressed (DE) genes based on a competing endogenous RNA (ceRNA) network is still currently lacking.

**Methods:**

In this study, we have systematically analyzed the gene expression profile data of neural progenitor cells (NPCs) derived from patients with HD and controls (healthy controls and the isogenic controls of HD patient cell lines corrected using a CRISPR-Cas9 approach at the HTT locus) to screen out DE mRNAs and DE lncRNAs and create a ceRNA network. To learn more about the possible functions of lncRNAs in the ceRNA regulatory network in HD, we conducted a functional analysis of Gene Ontology (GO) and Kyoto Encyclopedia of Genes and Genomes (KEGG) and established a protein–protein interaction (PPI) network for mRNAs interacting with these lncRNAs.

**Results:**

We identified 490 DE mRNAs and 94 DE lncRNAs, respectively. Of these, 189 mRNAs and 20 lncRNAs were applied to create a ceRNA network. The results showed that the function of DE lncRNAs mainly correlated with transcriptional regulation as demonstrated by GO analysis. Also, KEGG enrichment analysis showed these lncRNAs were involved in tumor necrosis factor, calcium, Wnt, and NF-kappa B signaling pathways. Interestingly, the PPI network revealed that a variety of transcription factors in the ceRNA network interacted with each other, suggesting such lncRNAs may regulate transcription in HD by controlling the expression of such protein-coding genes, especially transcription factors.

**Conclusions:**

Our research provides new clues for uncovering the mechanisms of lncRNAs in HD and can be used as the focus for further investigation.

## Background

Huntington's disease (HD) is an autosomal dominant-inherited polyglutamine disorder. Typical features include progressive movement disorder, neuropsychiatric problems, and cognitive impairment. Abnormal amplification of cytosine–adenine–guanine (CAG) repeats of the first exon in the huntingtin (*HTT*) gene encoding an abundantly-expressed 3144 amino acid protein, is the root cause of this fatal disease [1]. The DNA of healthy persons contains a region of less than 36 CAG repeats that encodes a polyglutamine (polyQ) tract in the huntingtin gene. An extension in the length of the polyQ tract at the N-terminal of the mutant huntingtin protein (mutHTT), encoded by an abnormal *HTT* gene containing an expansion of CAG repeats (> 36 in length), changes the conformation of mutHTT and leads to intracellular protein aggregates [2]. Carriers with longer CAG repeats of *HTT* exhibit different severities of HD, depending on the unusual length of CAG repeats [3]. How this pure mutant of the ubiquitously expressed protein leads to specific neurodegeneration is, however, unclear.

Over the past few decades, along with identifying the *HTT* gene, considerable progress has been made in learning the mechanism of neurodegeneration caused by mutHTT, including increased excitotoxicity injury in neurons through a deficiency of wild-type *HTT* [4], nutritional support disorders from neurons [5], the appearance of inclusion bodies derived from aberrant protein accumulation and the influence of polyglutamine length on the extent of mutHTT aggregates [6], mitochondrial impairment [7], and defective axonal transport in HD neurons [8, 9]. In addition to such cellular dysfunctions, mutHTT also caused complex transcriptional and post-transcriptional changes in mouse models and the brains of patients with HD, particularly in the striatum, followed by the cortex and cerebellum; the severity of transcriptional changes reflected the seriousness of neurodegeneration [10, 11]. Dysregulation of the RE1 silencing transcription factor (REST) [12] and its target gene regulatory networks, including microRNA (miRNAs) mir-132 [13], mir-9 [14], mir-124 [15] and possibly other non-coding RNAs (ncRNAs), was particularly attractive as an explanation of the neurodegeneration caused by mutHTT in related studies.

Recently, increasing studies have investigated the role of transcriptional and post-transcriptional dysregulation in the pathogenesis of HD, including a variety of both protein-coding and ncRNAs. In particular, the dysregulation of the protein-coding RNA, REST, in HD has become attractive. As a subtype of ncRNAs, long non-coding RNAs (lncRNAs) are a class of transcripts containing more than 200 bases that do not have a functional open reading frame, and play crucial biological roles in epigenetics and transcriptional regulation [16]. In addition to being involved in normal physiological processes, previous studies have shown that lncRNAs take part in the pathogenesis of HD by regulating the expression of protein-coding genes via both cis- and trans-action pathways [17]. They also interact with varying repressive chromatin regulatory complexes (PRC2, RCOR1, and SMCX), and affect transcription factor function [18, 19]. MicroRNAs are endogenous ncRNAs, approximately 22 nucleotides in length, that are involved in the negative regulation of mRNA at the post-transcriptional level by targeting a repressive protein complex (RISC) and pairing to the 3′-untranslated region of mRNA to directly repress post-transcriptional translation [20]. Increasing evidence has illustrated how several neuronal-specific miRNAs were dysregulated in HD disease [12], some of which were targeted by REST. In 2011, the competition endogenous RNA (ceRNA) hypothesis was proposed that emphasized how mRNAs, transcribed pseudogenes, and lncRNAs could interact with each other through competitive binding to miRNA response elements (MREs) [21]. It is therefore necessary to establish an lncRNA–miRNA–mRNA–ceRNA regulatory network in HD.

Increasing evidence has shown that lncRNAs, as well as ceRNAs, were associated with HD [17]. Clarifying the changes of the lncRNA regulatory network in HD neurons may perhaps help to identify and manage this intractable neurodegenerative disease. In this study, we acquired lncRNA and mRNA expression profile data of neural progenitor cells (NPCs) that were differentiated from HD (including corrected HD by a CRISPR-Cas9 approach) and healthy human-induced pluripotent stem cells (iPSCs) in the Gene Expression Omnibus (GEO) database. We then constructed a ceRNA regulatory network using differentially expressed genes (DEGs) that may help us to shed light on the mechanism of transcriptional changes in HD neurons.

## Methods

### Data acquisition and pre-processing

The expression dataset used in this study was collected from a previous study of NPC samples generated by HD human iPSCs acquired from the GEO database (https://www.ncbi.nlm.nih.gov/geo/) on the NCBI website. Series Matrix Files of GEO Series Accession NO.GSE93767 and the corresponding platform file of GPL10558 using an Illumina HumanHT-12 v4 Expression BeadChip (Illumina Inc., San Diego, CA, USA) were downloaded. This experiment was designed to explore transcriptional differences between HD and control cell lines, both of them derived from the HD (CAG180) / healthy (CAG33) human iPSCs and hiPSC-derived NPCs (the parental cell types were derived from human fibroblasts), and to identify genome-wide molecular changes after correction of the mutation in the HD cell line using a CRISPR/Cas9 and piggyBac transposon-based approach [22]. A Cas9 nickase (Cas9n)-mediated cleavage of CAG180 iPSCs was carried out using a pair of selected small-guide RNAs at the *HTT* locus to correct the disease mutation. A piggyBac transposon selection cassette-based homologous recombination donor was used to establish isogenic control lines. After genome editing and targeted clones screening, a whole-exome sequencing was performed that identified a low off-target activity in a comparison of the isogenic control iPSCs and their parental CAG180 line. All of these cell lines were then efficiently differentiated into forebrain NPCs [22]. In this paper, two corrected isogenic controls of CAG180 HD NPC cell lines (HD-C#1,2), and the non-isogenic CAG33 healthy NPC cell line, were included in the control group; the non-corrected CAG180 HD NPC cell line was included in the HD group. A total of 12 samples were used for this analysis, with three replicates per cell line per cell type. Expression values in the expression matrix file were the log-converted and adjusted data [22].

Due to the probes in the HumanHT-12 v4 Expression BeadChip derived from an earlier version of the NCBI Reference Sequence (RefSeq), RNAs Release 38, we re-matched all 48,107 probe sequences to human NCBI RefSeq RNA Release 109 using a Basic Local Alignment Search Tool (optimized for highly similar sequences) from NCBI, 38,640 probes were retained. All probes were then converted to their corresponding gene symbols. Before differential expression analysis, if a gene symbol matched more than one probe, mean processing of the data was undertaken for all corresponding probes. We then analyzed these expression values using an R-Studio tool (version 1.1.463). All expression values were normalized by quantile normalization in package limma [23]. To discover which genes were targeting lncRNAs or protein-coding genes, we remapped these genes to the human reference genome annotation file downloaded from NCBI (Homo_sapiens.GRCh38.94.chr.gtf); genes without corresponding annotation information were removed. After the above filtering, 20,897 genes in the final dataset remained.

### Differentially expressed genes and construction of lncRNA-associated ceRNA network

**Fig. 1:**
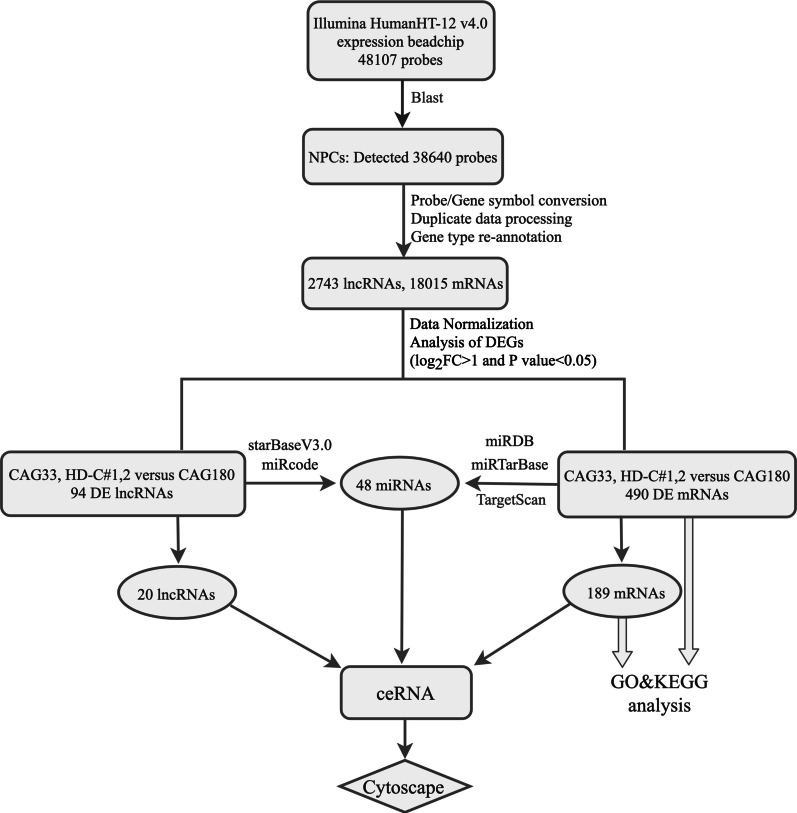
Flow diagram of the data analysis

Differential expression analysis was performed in R studio software version 1.1.463 [24] using a Bioconductor 3.8 package (http://www.bioconductor.org/), with an absolute log_2_^fold−change^ (|logFC|) > 1 and *P* < 0.05 as cutoff values by using an empirical Bayes method for screening DE lncRNAs and mRNAs. A lncRNA-related ceRNA regulatory network was constructed according to the "ceRNA hypothesis", in which lncRNA can regulate the expression of mRNAs by competitively binding miRNA, which contains common MREs. Therefore, the miRNAs targeted to DE lncRNAs were predicted by starBase v3.0 based on Ago CLIP-sequencing (seq) data with a threshold of high stringency (≥ 3) [25] or by miRcode based on highly conserved miRNA targets [26]. Next, the DE mRNAs targeted-miRNAs were predicted by at least by two or more databases among miRDB [27], TargetScanHuman 7.2 [28], and the experimentally validated miRNA-target interactions database miRTarBase 7.0 [29]. Based on the acquired DE lncRNAs, DE mRNAs, and their co-targeted miRNAs, a ceRNA regulatory network was established and depicted by Cytoscape v3.7.0 software [30]. A flow diagram (Fig. 1) clearly shows how we undertook data analysis.

### PPI network analysis

To better identify critical genes and clarify the potential relationships of mRNAs in the ceRNA network, protein–protein (PPI) network analysis was carried out using STRING software (score > 0.4) [31].

### GO, KEGG functional enrichment analysis

To clarify which biological processes and pathways of NPCs induce significant changes in HD for all aberrantly expressed protein-coding RNAs and those included in the ceRNA network, we performed functional enrichment analysis of GO using R software [32]. We created and visualized a functional enrichment network of KEGG pathways using Cytoscape plug-in ClueGO v2.5.4 [33] and CluePedia v1.5.4 [34] with the human genome as a background. The ClueGO network was built using kappa statistics to reflect the relationship between terms according to the resemblance of related genes. Functionally grouped networks with terms as nodes were connected based on their kappa score level (≥ 0.4). Pathways showing a *p*-value < 0.05 were regarded as remarkably enriched for both GO and KEGG analyses.

## Results

### Probe re-annotation

A total of 38,640 probes were re-annotated into corresponding gene symbols, and after deduplication using a mean method and data normalization, a final set of 2743 lncRNAs and 18,015 mRNAs was retained in the matrix file for subsequent analysis.

### Identification of differentially expressed genes

**Fig. 2:**
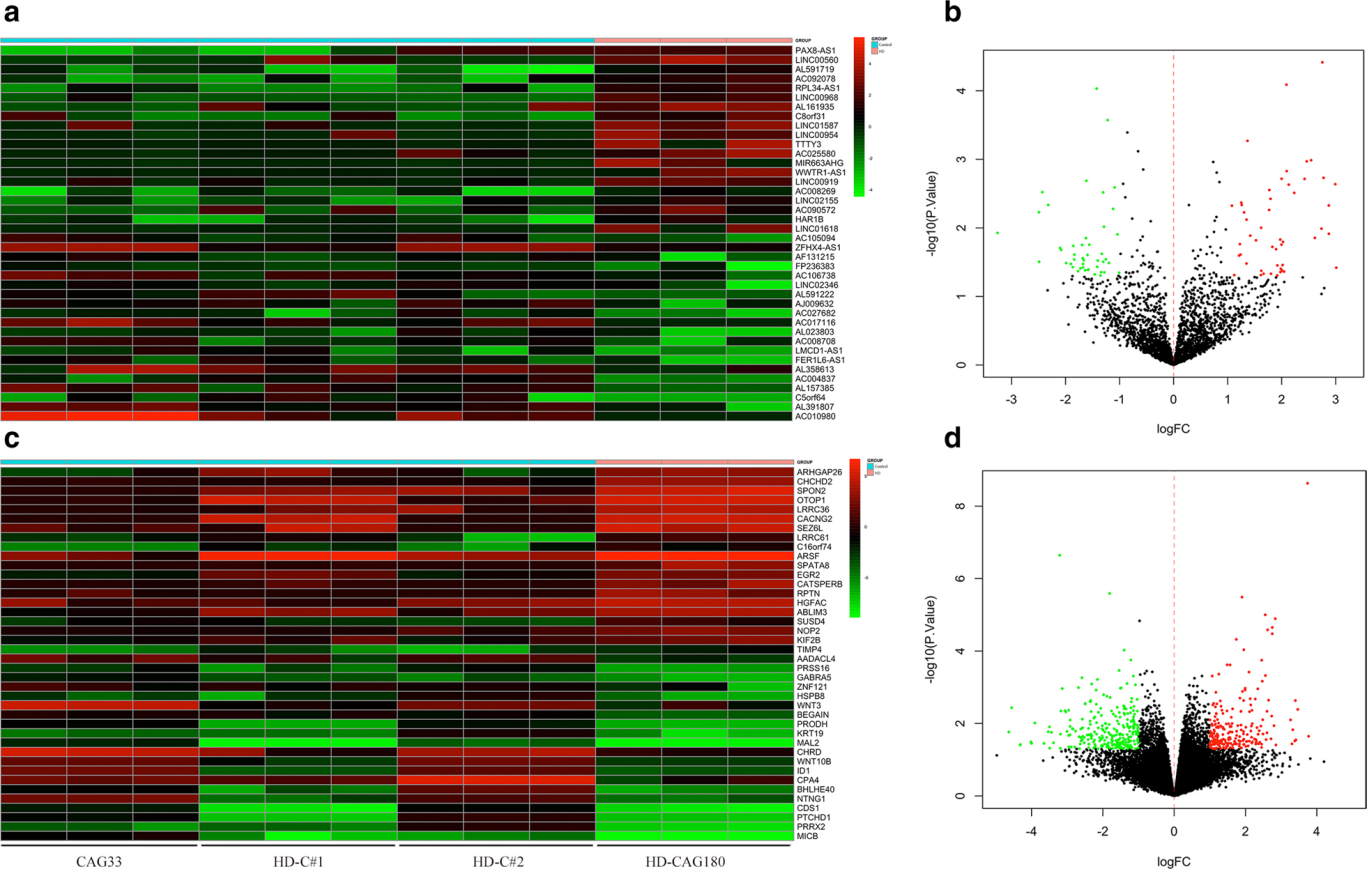
DEGs analysis and visualization. **a**, **c**. Heat map of the top 20 differentially expressed (DE) long non-coding RNAs (lncRNAs) and mRNAs. **b**, **d**. Volcano plots of lncRNAs and mRNAs. Red and green represented up- and down-regulated differentially expressed genes (DEGs), respectively

Compared with the control group (CAG33 and HD-C#1, 2), a total of 94 DE lncRNAs (49 up-regulated, 45 down-regulated) and 490 DE mRNAs (229 up-regulated, 261 down-regulated) were found in HD cell lines with 180 CAG repeats by differentially expression analysis. The strength of differential gene expression was shown in the form of volcano plots, and the top 20 up/down-regulated DE lncRNAs and mRNAs were represented by heatmaps, respectively (Fig. 2).

### Function analysis of DE mRNAs

**Fig. 3:**
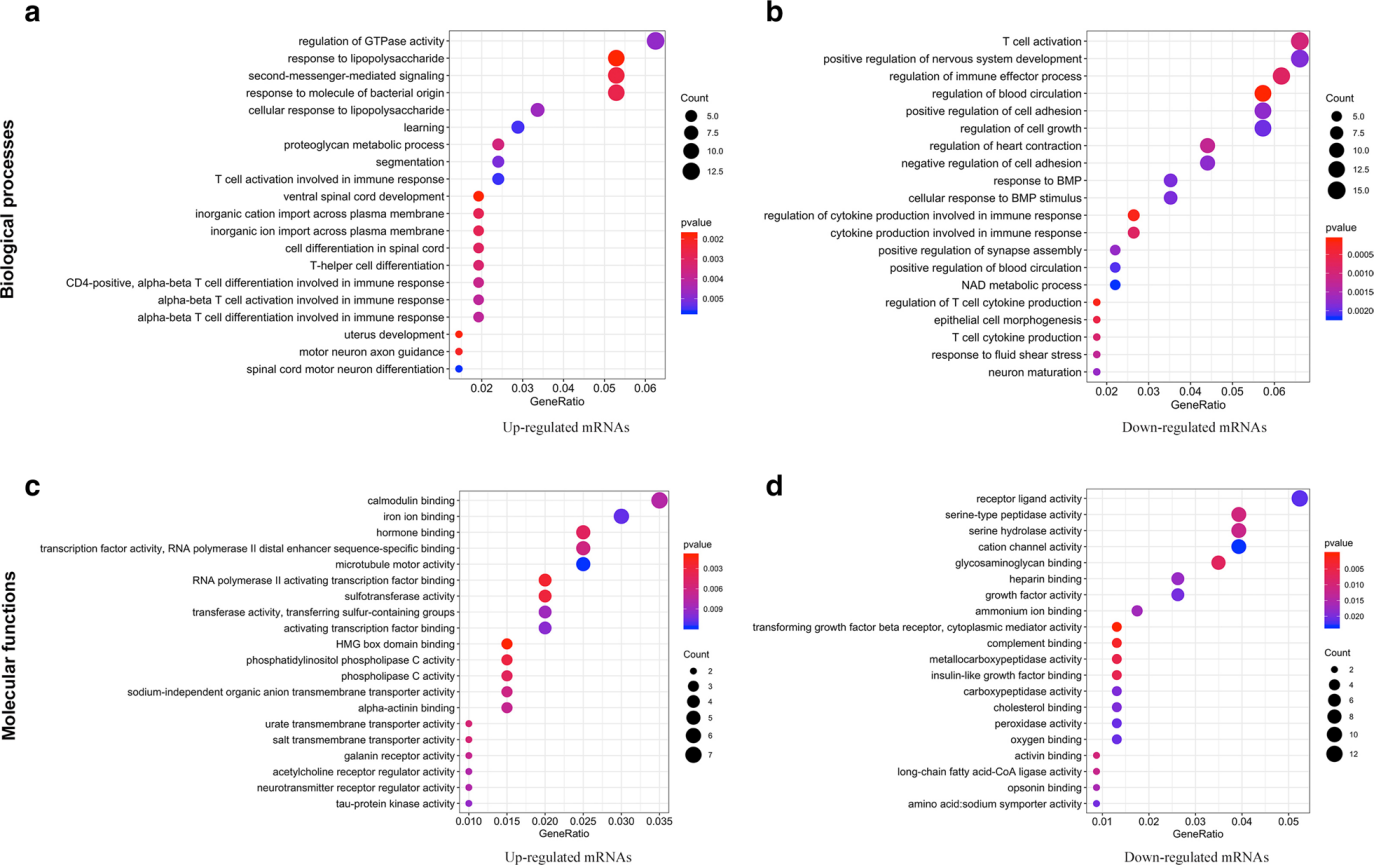
Top 20 significantly enriched GO categories of DE mRNAs. Biological processes (**a**, **b**) and molecular functions (**c**, **d**) of aberrantly expressed protein-coding RNAs are shown in the pattern of dot plots. The size of the solid dots represents gene counts, and the colors from red to blue correspond to *p*-values from small to large. GO, Gene Ontology; DE, differentially expressed

We further investigated the biological functions of up- and down-regulated protein-coding mRNAs using Gene Ontology (GO) and Kyoto Encyclopedia of Genes and Genomes (KEGG) analyses. As shown in Fig. 3, the top 20 biological processes (BPs) and molecular functions (MFs) of protein-coding RNAs were exhibited as dot plots. We found that the significant changes in BPs of up-regulated mRNAs included the regulation of GTPase activity, a response to lipopolysaccharide, second messenger-mediated signaling, immune responses, spinal cord, and neurodevelopment and differentiation. For down-regulated mRNAs, their BPs included T-cell activation, the positive regulation of nervous system development and cell adhesion, the response to bone morphogenetic protein (BMP), the immune response, T cell cytokine production, and neuron maturation. Similarly, the significant MF changes of up-regulated mRNAs included calmodulin binding, transcription factor activity, RNA polymerase II distal enhancer/proximal promoter sequence-specific DNA binding, iron ion binding, phospholipase C activity, and transmembrane transporter activity. Receptor ligand activity, channel activity, serine-type peptidase/hydrolase activity, and transcriptional activator activity were related to the MFs of down-regulated mRNAs.

**Fig. 4:**
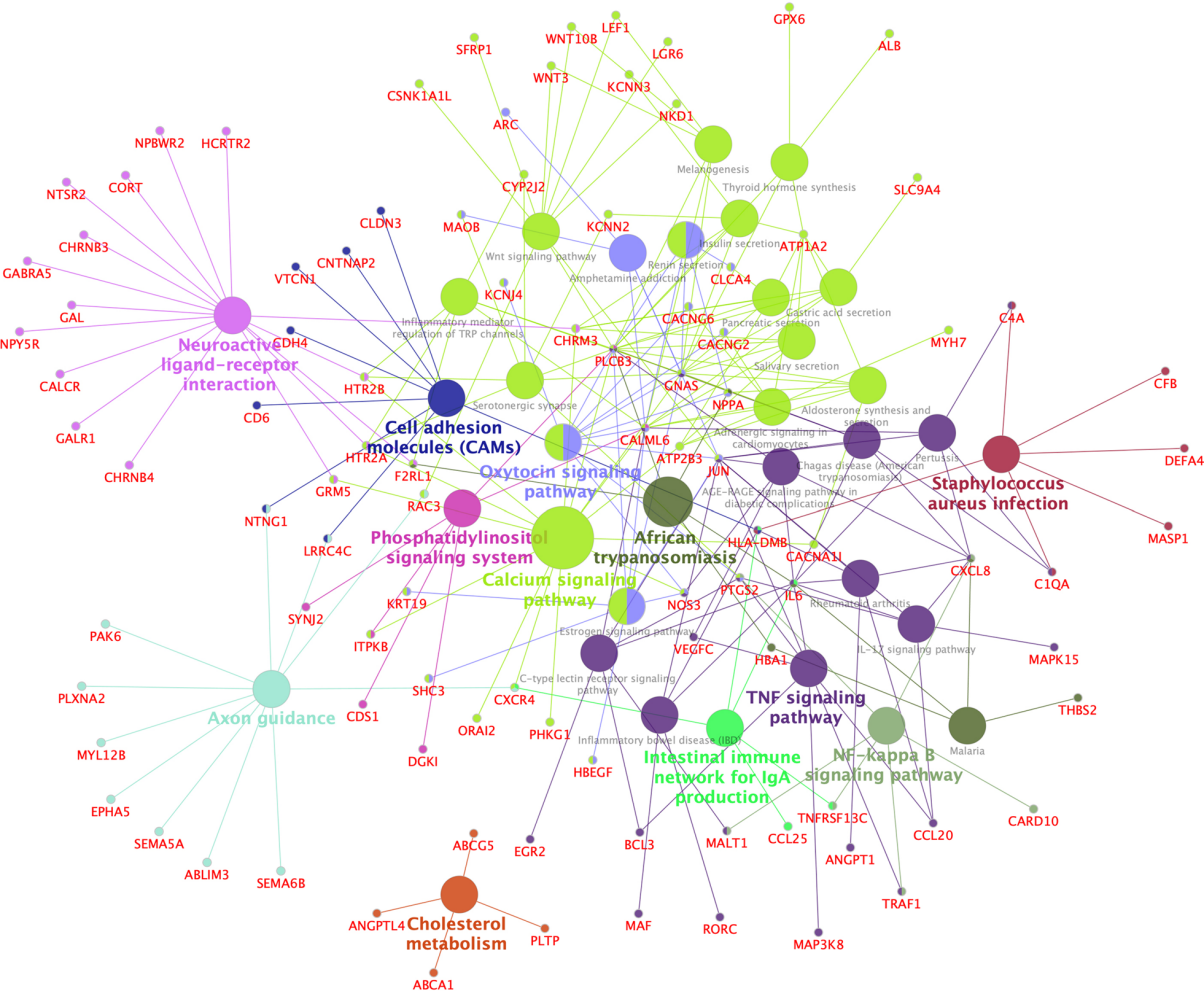
KEGG Pathway Enrichment Analysis of DE mRNAs. The results of Kyoto Encyclopedia of Genes and Genomes (KEGG) analysis acquired by ClueGO/CluePedia software were visualized by a Cytoscape platform, with *p* < 0.05 as the cutoff value. Terms grouped with a kappa score level (≥ 0.4) are shown as nodes of different colors. The size of each node represents significances whereas only the labels of the most critical terms per group are shown in colors. Edges link small nodes representing genes that are related to the corresponding terms. DE, differentially expressed

To identify which pathways were involved in the DEGs, all DE mRNAs were matched to pathways in the KEGG database, and 34 significantly enriched pathways were identified (*p* < 0.05; Fig. 4). When functionally grouped based on shared genes between terms with a kappa score level (≥ 0.4), 12 functional groups were obtained in the final analysis, such as the calcium signaling pathway, tumor necrosis factor (TNF) signaling pathway, cell adhesion molecules, and NF-kappa B signaling pathway.

### LncRNA-associated ceRNA network

**Fig. 5:**
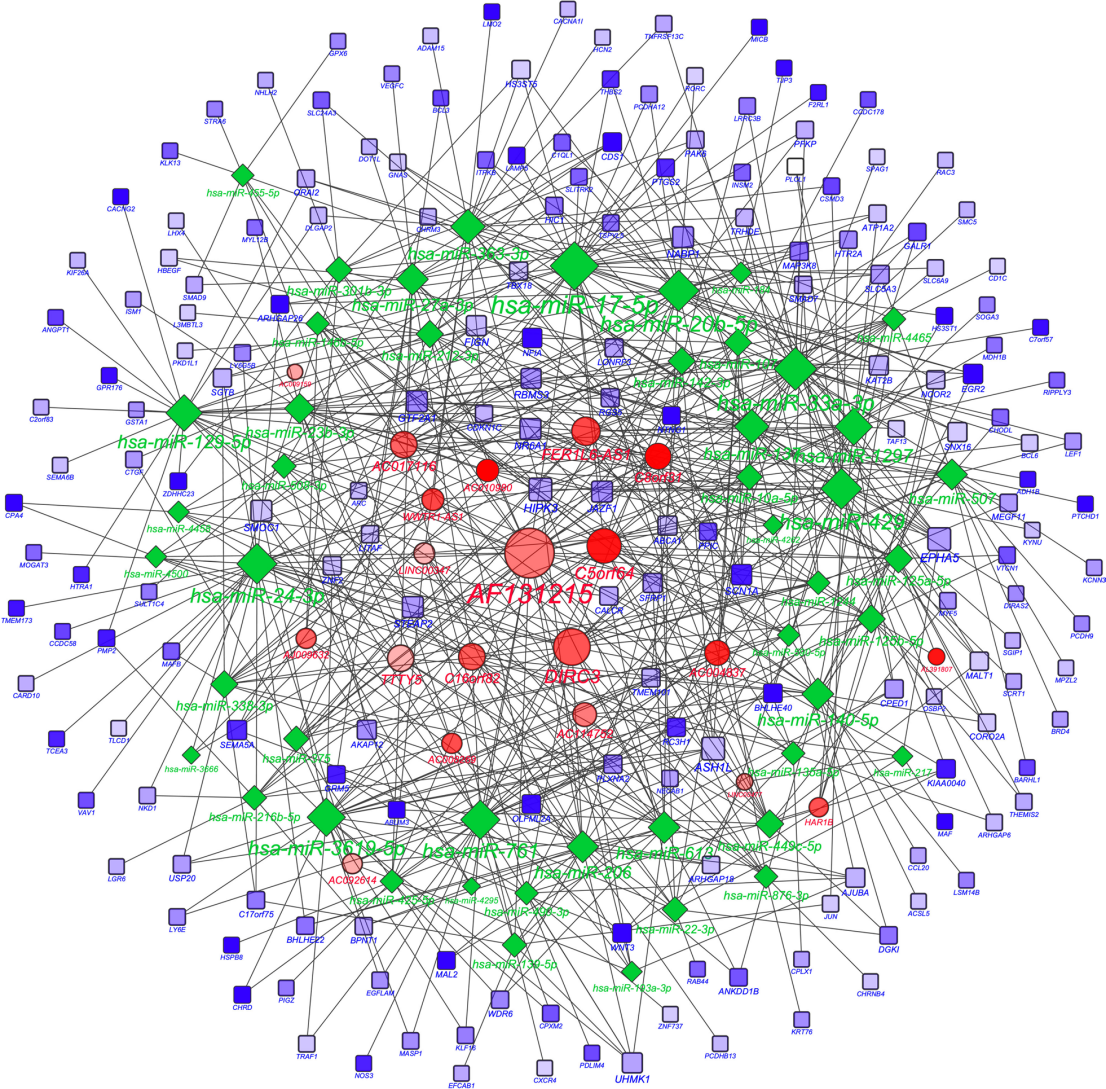
A view of the ceRNA network. Long non-coding RNA (lncRNA), mRNA, and micro(mi)RNA are defined by red circles, blue squares and green rhombuses, respectively. A total of 20 lncRNAs, 48 miRNAs, 189 mRNAs, and 687 edges were included in the competing endogenous RNA (ceRNA) network. The size of each node represents the number of connected edges. The transparency of the color represents the significance of the differences; the more in-depth the color, the more significant of the differences

As miRNA sponges, lncRNAs can isolate and bind miRNAs to regulate mRNA expression. By using the predictive databases of starBase, miRcode, miRDB, TargetScanHuman, and miRTarBase, we identified 48 common miRNAs that were targeted by both 20 differentially expressed lncRNAs and 189 mRNAs. In accordance with these data, we constructed an lncRNA–miRNA–mRNA competitive regulation network (Fig. 5). The top 10 lncRNAs with the highest connectivity are listed (Table 1).

**Table 1:** Top 10 genes with the highest connectivity in the ceRNA regulatory network

lncRNA	Degree of connectivity	miRNAs	Degree of connectivity	mRNAs	Degree of connectivity
AF131215	38	hsa-miR-17-5p	36	ASH1L	9
DIRC3	24	hsa-miR-33a-3p	30	HIPK3	9
C5orf64	21	hsa-miR-429	28	EPHA5	9
FER1L6-AS1	15	hsa-miR-20b-5p	28	NR6A1	7
AC017116	13	hsa-miR-24-3p	27	STEAP2	7
C16orf82	13	hsa-miR-761	26	SMOC1	7
TTTY5	13	hsa-miR-3619-5p	25	JAZF1	7
C8orf31	12	hsa-miR-1297	24	GTF2A1	7
AC004837	11	hsa-miR-129-5p	23	NABP1	7
AC114752	10	hsa-miR-363-3p	21	FIGN	6

*lncRNA* long non-coding RNA, *ceRNA* competing endogenous RNA, *miRNA* microRNA

### Construction of PPI network and GO/KEGG analysis of the mRNAs interacting with lncRNAs in the ceRNA network

**Fig. 6:**
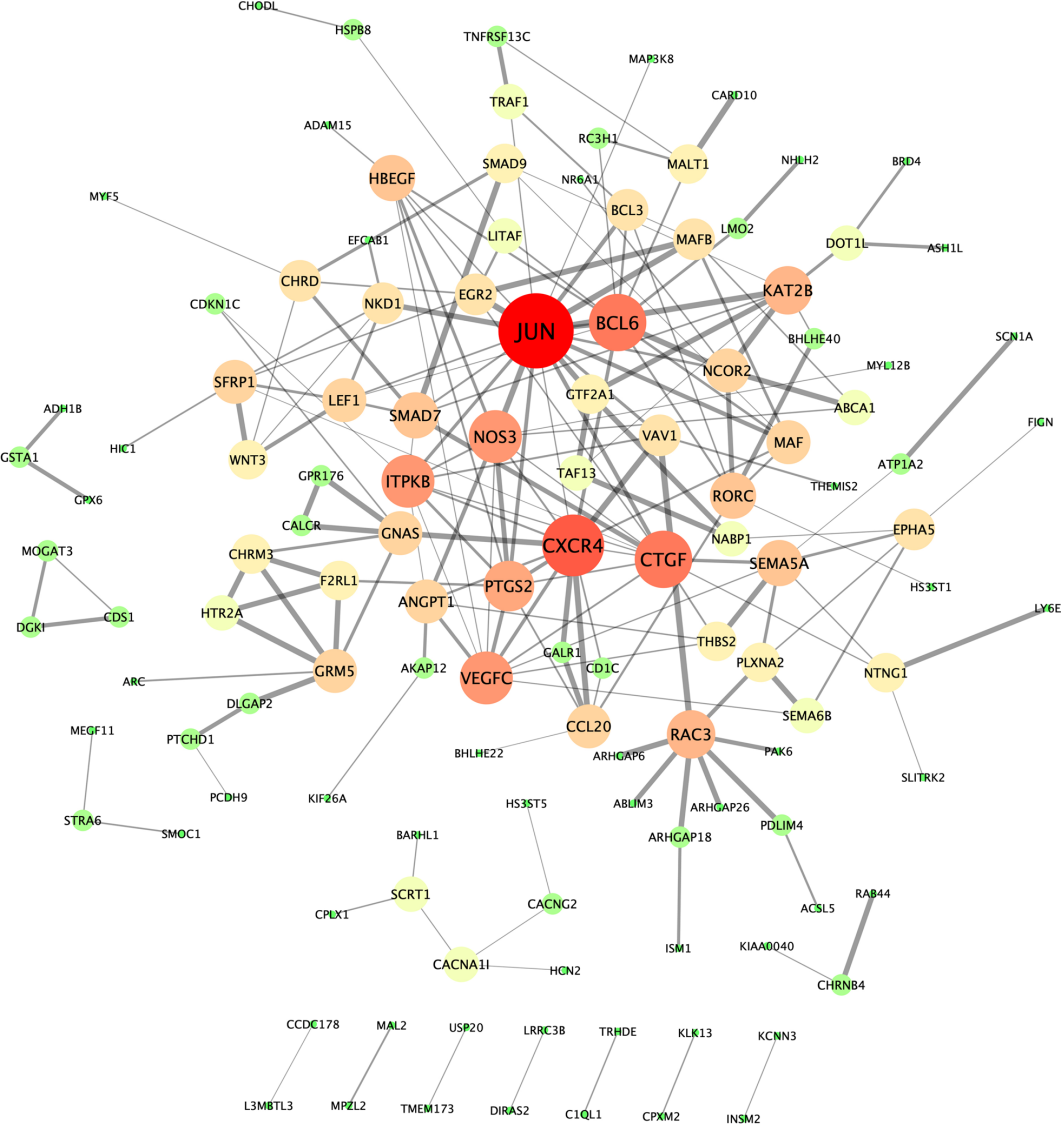
The protein–protein interaction network. This protein–protein interaction (PPI) network comprises of 123 mRNAs with an interaction score > 0.4 in STRING software that was optimized with Cytoscape software. The mRNAs are shown in different sizes and gradient colors of nodes based on the degree of edges. Green, yellow, or red correspond to low, medium, or high degrees, respectively. The edge thickness indicates interaction scores

To better identify critical genes and understand the biological function of mRNAs in the ceRNA network, we established a protein–protein interaction (PPI) network in STRING software [31] with interaction scores of > 0.4 (Fig. 6) and performed GO enrichment analysis using the background of a human whole-genome (Tables 2, 3). As the PPI network showed, the primary hub nodes were JUN, CXCR4, BCL6, CTGF, NOS3, VEGFC, ITPKB, PTGS2, RAC3, and KAT2B. Through an enrichment analysis of their protein interacting partners, we observed that the most significant biological processes defined by GO were the response to stimulus (GO:0048583), regulation of signaling (GO:0023051), cell communication (GO:0010646), and signal transduction (GO:0009966), and positive regulation of biological process (GO:0048518). About half of the significant molecular functions were related to transcriptional regulation (GO:0000976, GO:0000977, GO:0003700, GO00140110).

**Table 2:** Top five terms for Biological Process (GO) of predicted mRNAs in ceRNA

GO-term	Description	Count in a gene set	False discovery rate
GO:0048583	Regulation of response to stimulus	71/3882	2.53E-05
GO:0023051	Regulation of signaling	65/3360	2.53E-05
GO:0010646	Regulation of cell communication	65/3327	2.53E-05
GO:0009966	Regulation of signal transduction	58/3033	0.00013
GO:0048518	Positive regulation of biological process	84/5459	0.00070

*GO* Gene Ontology, *ceRNA* competing endogenous RNA

**Table 3:** Top 5 terms of molecular function (GO) of predicted mRNAs in ceRNA

GO-term	Description	Count in a gene set	False discovery rate
GO:0140110	Transcription regulator activity	36/2069	0.0389
GO:0044212	Transcription regulatory region DNA binding	19/829	0.0389
GO:0043167	Ion binding	84/6066	0.0389
GO:0005515	Protein binding	86/6605	0.0389
GO:0005488	Binding	139/11,878	0.0389

*GO* gene ontology, *ceRNA* competing endogenous RNA

**Fig. 7:**
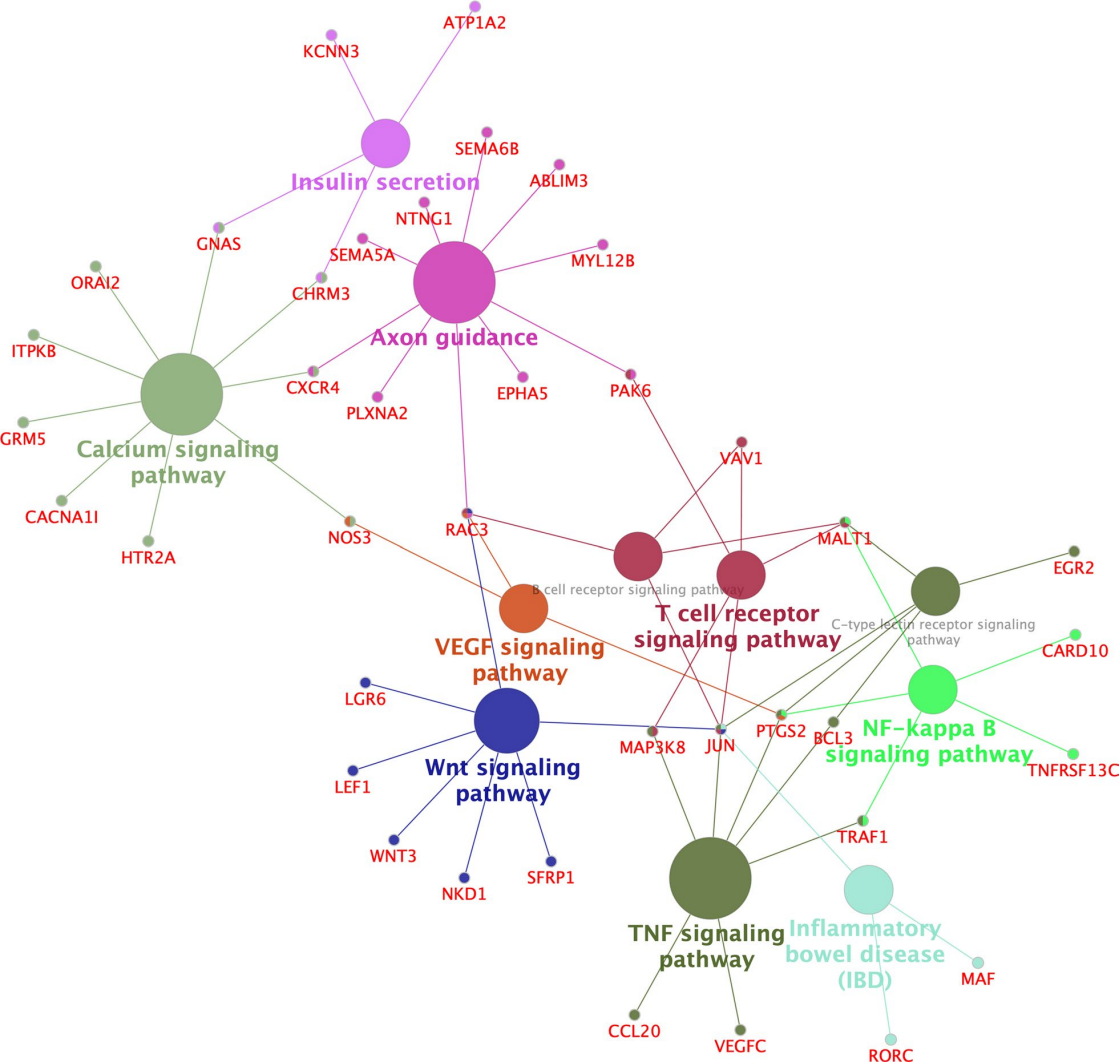
A view of KEGG Pathway Enrichment Analysis of mRNAs in ceRNA. The Kyoto Encyclopedia of Genes and Genomes (KEGG) pathway enrichment graph was visualized by Cytoscape software based on ClueGO/CluePedia KEGG analysis with *p* < 0.05 and a kappa score ≥ 0.4. See Fig. 3 for the meaning of nodes and edges. ceRNA, competing endogenous RNA; VEGF, vascular endothelial growth factor; TNF, tumor necrosis factor

Further, KEGG enrichment analysis based on ClueGO/CluePedia with *p* < 0.05 and a kappa score ≥ 0.4 revealed that the top five significant enrichment pathways were axon guidance, and the signaling pathways of TNF, calcium, Wnt, and NF-kappa B (Fig. 7).

## Discussion

Emerging evidence over decades has revealed that ncRNAs play a vital role in neuronal development and function, by modulating DNA replication, epigenetic modifications, controlling the transcriptional and post-transcriptional process, and by regulating miRNA activity and mRNA translation and protein stability [35–37]. Studies have shown that lncRNAs are important and poorly explored molecules related to neurological disease [38] involving Parkinson’s Disease (PD), Alzheimer’s Disease (AD), fragile X syndrome, and spinocerebellar ataxia [39–41]. Over the past few years, several lncRNAs, including HTT-AS [42], BDNF-AS [43], ABHD11-AS1 [44], neural human accelerated region 1 (HAR1) [45], TUNA [46], and NEAT1 [47], among others, have been found to be related to the altered expression of HD-related genes, and changes in these have contributed to neuronal apoptosis and the increased mHTT toxicity observed in HD. Moreover, microarray data analysis from HD brain tissues has also revealed multiple misregulated lncRNAs such as TUG1, LINC000341, RPS20P22, MEG3, DGCR5, and LINC000342, among others [17]. However, most HD-related lncRNAs with known functions mainly act as epigenetic or transcriptional regulators in the cellular nucleus; however, cytoplasmic processes remain elusive.

Studies have revealed that some lncRNAs acting as "miRNA sponges" that compete with the miRNA target gene sponge and bind MREs, which alleviates miRNA-mediated target mRNA inhibition and subsequently promotes miRNA degradation through double-stranded RNA formation [48]. This ceRNA regulatory mode is one of the essential ways for ncRNAs to regulate biological functions by interacting with their downstream target mRNAs. Here, we have identified hundreds of DE lncRNAs and DE mRNAs in HD NPC cell lines, using a bioinformatics analysis method widely used in experimental and clinical studies to identify DEGs and pathways, diagnostic biomarkers, and potential therapeutic agents [49], and to determine the genetic basis of several diseases [50]. However, most of DE lncRNAs have not been investigated in previous studies. Also, we created a ceRNA regulatory network to explore their potential pathways and possible roles in HD. We found that the most significant molecular function of mRNAs in the ceRNA network was involved in transcriptional regulation; extensive transcriptional disorder has been shown to be an early process of HD in cells and animal models, as well as in postmortem HD brain tissues [11, 51, 52]. Here, we speculate that lncRNAs in the ceRNA network may be involved in the regulation of transcription. Intriguingly, in the present study, lncRNA-HAR1B, which was previously shown to be down-regulated in HD patients, was also shown to be dysregulated in our study. It is down-regulated in the striatum of patients with HD, owing to repression by its direct target, REST, a critical neural gene regulator [45]. However, currently, little is known about the mechanism or function of HAR1B. Another significantly dysregulated lncRNA, paired box 8 (PAX8) antisense RNA1 (PAX8-AS1), is a potential regulator of PAX8 [53], which is linked to cell cycle control and metabolic processes [54]; however, its roles or mechanisms in HD remains unknown. The dysregulated lncRNA-chromosome 5 open reading frame 64 (C5orf64), of which the expression level is positively correlated with tumor-infiltrating immune cells including M2 macrophages, monocytes, eosinophils and neutrophils, can function by ceRNA network and be closely correlated with the tumor microenvironment in lung adenocarcinoma [55]. However, their functionals and roles in HD need to be further explored.

Multiple aberrant molecules observed here may contribute to the pathogenesis of HD in various ways. The transcription factor, JUN, also known as c-Jun and AP1, is markedly dysregulated in our ceRNA network and is a crucial target of the c-Jun NH2-terminal protein kinase (JNK) pathway. As an essential mediator of apoptosis in different model systems, JNK is implicated in the regulation of biological processes at both transcriptional and post-transcriptional levels [56]. JNK is activated in HD so that its inhibition may be beneficial in correcting HD-correlated neurotoxicity [57]. From our results, other transcription factors in our results, such as KAT2B, EGR2, MAFB, and GTF2A1, which may be dysregulated through ceRNA network interaction, contribute to transcriptional activation and interact with JUN in the PPI network. Other molecules, for example, JUN, LGR6, SFRP1, WNT3, NKD1, RAC3 and LEF1 in the ceRNA network, might be involved in the canonical Wnt/β-catenin signaling pathway (Fig. 7). As we know, the Wnt/β-catenin signaling pathway is an important component in the development of many neurodegenerative diseases [58, 59]. In HD models, mutHTT interferes with β-catenin degradation by binding to several components of the β-catenin degradation complex, This results in an abnormal accumulation of cytoplasmic β-catenin that cannot enter the cellular nucleus to activate the transcription of pro-survival target genes [60], leading to excessive neuronal apoptosis [61]. Therefore, further studies are needed to evaluate other molecules, as mentioned above, involved in this critical pathway in HD.

In addition to transcriptional dysregulation, neuroinflammation is also one of the typical features of most neurodegenerative diseases, including HD [62, 63]. Previous studies have observed elevated levels of various pro-inflammatory cytokines in the blood and brain tissues of mice and patients with HD, suggesting that inflammation may contribute to HD progression; some inflammatory cytokines are related to the TNF signaling pathway [64–66]. Moreover, the NF-κB signaling pathway, by modulating the cytokine production, plays a crucial role in inflammation in HD. The overexpression of mutHTT can activate the NF-κB pathway by directly interacting with a critical regulator of NF-κB, the IkappaB kinase complex; this may contribute to neurodegeneration [67]. Additionally, increased activation of NF-κB has been found in astrocytes of HD patients and in a mouse model of HD, while an inflammatory response mediated by NF-κB in astrocytes facilitates the pathogenesis of HD [68]. In our study, we observed how several dysregulated mRNAs, such as TRAF1, PTGS2, and MAP3K8, were involved in the inflammatory response by TNF and NF-κB signaling pathways (Fig. 7). This can affect a wide range of functions such as apoptosis and cell survival, as well as inflammation and immunity.

A calcium signaling pathway is critical to neuronal function. In neurodegenerative diseases, impaired Ca2+ signal transduction can interfere with mitochondrial function and synaptic plasticity [69]. The impairment of mitochondrial function and alteration of intracellular calcium-induced calcium release and blockade mechanisms exacerbate damage in the loop function of HD [70]. Reduced Ca2+ levels in the endoplasmic reticulum and enhanced store-operated calcium entry channels, which play an essential signaling function in neurons [71], lead to a synaptic decline in HD [69]. Dysregulation of *GNAS*, *CXCR4*, *ORAI2*, *ITPKB*, and *CACNA1I* genes observed here in the calcium signaling pathway seems related to the pathogenesis of HD and needs to be further elucidated in future.

Here, we also found that many axon guidance–related genes are contained in the ceRNA network. Nevertheless, few studies have involved in the correlation between axon guidance and HD. Axon guidance is essential for intricate neural circuit formation in brain development [72]. Some studies have shown an abnormal expression or mutation of axon guidance–related genes or proteins that are indispensable for maintaining normal synaptic structure and connections in adults, and are associated with different neurological disorders [72, 73], such as AD, PD, autism spectrum disorder (ASD), amyotrophic lateral sclerosis as well as other diseases. For example, netrin-1 plays a protective role against AD and may be a potential target for AD [74, 75]. Mice with a deficiency in the *SEMA5A* gene presented with ASD-like behaviors [76]. A mutation of *SEMA5A* was found in a patient with ASD [77]. In HD, synaptic dysfunction is one of the typical pathogenic features, based on our results and the role of axonal guidance in other neurodegenerative diseases, and it is reasonable to believe that the aberrant axonal guidance may participate in the pathogenesis of HD. Nevertheless, relevant knowledge is limited, and further studies are needed to clarify this problem.

While the functions and mechanisms of most DE lncRNAs here remain unclear, they may play a role in the pathogeneses of HD by controlling the expression of the above-mentioned coding genes in the ceRNA network. In future, further in vitro and in vivo experiments are needed to verify the ceRAN regulatory network established here and to elucidate its roles in HD pathogenesis.

The limitations of this study should also be mentioned. First of all, we must be clear that the differentially expressed molecules screened using various bioinformatics methods only represent a certain disease stage, while changes in gene expression is an intricate and dynamic process rather than existing in a stationary system. Second, lncRNAs can play a role in different ways by virtue of their nuclear or cytoplasmic localization. Here, we only explored the role of lncRNAs as miRNA sponges in the ceRNA network in NPC cells derived from iPSC cell lines. Third, only 12 samples were used in this study, with a comparison made between a CAG33 healthy controls and CAG180 HD cell lines. In addition, as with the results acquired through various bioinformatics methods, the DE lncRNAs we obtained here need to be further validated in HD cells or animal models, including their potential expression changes and functions. Finally, the upstream regulatory molecules of lncRNAs have not been explored, and the relationship between mutHTT and the lncRNAs within this study remains to be elucidated.

## Conclusions

In conclusion, by using bioinformatics methods in the present study, we have successfully identified hundreds of DE mRNAs and lncRNAs that were not previously linked to HD. By creating a ceRNA network with differentially expressed coding genes and lncRNAs, and in predicting miRNAs, it was revealed that lncRNAs may be involved in facilitating the expression of corresponding mRNAs through an lncRNA–miRNA–mRNA regulatory mechanism, contributing to the pathological processes of HD. Functional and PPI network analyses demonstrated that a variety of transcription factors were dysregulated, the expression of which were controlled by lncRNAs. It is further suggested that the function of lncRNAs may be related to a wide range of transcriptional regulation. This study provides new clues to uncovering the mechanisms of lncRNAs in HD and can be used as the focus for further investigation.

## Data Availability

The datasets used and/or analysed during the current study are available from NCBI Gene Expression Omnibus (GEO: GPL10558, GSE93767) https://www.ncbi.nlm.nih.gov/geo/.

## Abbreviations

HD: Huntington's disease
BP(s): Biological processe(s)
CAG: Cytosine–adenine–guanine
ceRNA: Competing endogenous RNA
DE/DEG(s): Differentially expressed/differentially expressed gene(s)
GO: Gene Ontology
HTT: Huntingtin
iPSC(s): Induced pluripotent stem cell(s)
KEGG: Kyoto Encyclopedia of Genes and Genomes
lncRNA(s): Long non-coding RNA(s)
MF: Molecular function
mutHTT: Mutant huntingtin
NPC(s): Neural progenitor cell(s)
polyQ: Polyglutamine
PPI: Protein–protein interaction
REST: Repressor element 1 (RE1) silencing transcription factor
RISC: Repressive protein complex
